# The Linoleic Acid: Dihomo-γ-Linolenic Acid Ratio (LA:DGLA)—An Emerging Biomarker of Zn Status

**DOI:** 10.3390/nu9080825

**Published:** 2017-08-01

**Authors:** Marija Knez, James C. R. Stangoulis, Maria Glibetic, Elad Tako

**Affiliations:** 1 College of Science and Engineering, Flinders University, GPO Box 2100, Adelaide, SA 5001, Australia; james.stangoulis@flinders.edu.au; 2 Centre of Research Excellence in Nutrition and Metabolism, Institute for Medical Research, University of Belgrade, 11000 Belgrade, Serbia; mglibetic@gmail.com; 3 USDA/ARS (US Department of Agriculture, Agricultural Research Service), Robert W. Holley Centre for Agriculture and Health, Cornell University, Ithaca, NY 14853, USA; et79@cornell.edu

**Keywords:** biomarker, Zn, LA:DGLA, Zn status, fatty acid

## Abstract

Zinc (Zn) deficiency is a common aliment predicted to affect 17% of the world’s population. Zinc is a vital micronutrient used for over 300 enzymatic reactions and multiple biochemical and structural processes in the body. Although whole blood, plasma, and urine zinc decrease in severe zinc deficiency, accurate assessment of zinc status, especially in mild to moderate deficiency, is difficult as studies with these biomarkers are often contradictory and inconsistent. Hence, as suggested by the World Health Organization, sensitive and specific biological markers of zinc status are still needed. In this review, we provide evidence to demonstrate that the LA:DGLA ratio (linoleic acid:dihomo-γ-linolenic acid ratio) may be a useful additional indicator for assessing Zn status more precisely. However, this biomarker needs to be tested further in order to determine its full potential.

## 1. Introduction

Zinc (Zn) deficiency was first described in humans in the early 1960s, in Middle Eastern, male adolescent dwarfs consuming plant-based diets [1]. Subsequently, Zn deficiency has been identified in many other regions of the world, and it became evident that dietary deficiency of Zn in humans is a widespread phenomenon. Today, Zn deficiency affects around 17% of the world’s population [2]. During the last 50 years, tremendous advances have been made both in our basic and clinical understanding of Zn metabolism [3]. Major progress has been made in understanding the importance of Zn as a structural and catalytic factor in a wide range of biological reactions, in uncovering the cellular Zn absorption and excretion mechanisms, and in clarifying the activities of major Zn transporters (15 Zip and 10 ZnT transporters) [4,5]. A significant research effort has tried to identify a physiological biomarker that predicts Zn status truthfully, especially in mild to moderate Zn deficiency. However, to this day, we are still without an entirely accurate biomarker of Zn status. In 2009, Lowe and colleagues evaluated 32 biomarkers in their review and identified only three as potentially useful: serum/plasma Zn concentrations, hair Zn concentration, and urinary Zn excretion [6]. Similarly, the BOND (Biomarkers of Nutrition for Development) Zinc Expert Panel recommended the following zinc biomarkers for use: dietary intakes, plasma/serum Zn concentrations, and stunting [7]. Last year, an update on Zn biomarkers was provided, recognizing a few as emerging biomarkers that require further investigation: Zn-dependent proteins, taste acuity, oxidative stress, and DNA integrity [8].

This review is a summary of research related to the LA:DGLA ratio (linoleic acid:dihomo-γ-linolenic acid ratio) as a novel biomarker of Zn status. We describe the chemical structure and function of the ∆6 desaturase enzyme, outline the current knowledge related to the role of Zn in desaturase activity and fatty acid metabolism, and provide recent data that demonstrates the usefulness of the LA:DGLA ratio to be used as a potentially new biomarker of Zn status. Lastly, we allude to further research needed on this topic.

## 2. The Limitations of the Currently Used Biomarkers: “Emerging” Biomarkers

All of the currently accepted and commonly used biomarkers of Zn status have certain limitations. Serum/plasma Zn, hair Zn concentration, and urinary Zn levels tend to fall in severe Zn depletion [9]. However, while plasma Zn concentration responds to altered intake over a short period of time, low plasma Zn concentrations do not remain constant for an extended period due to the homeostatic mechanisms that act to maintain plasma zinc concentration within the physiologic range; by maintaining losses via the GI (gastrointestinal) tract and kidneys [6]. Similarly, dietary Zn intake very often is not correlated with plasma zinc status, and does not realistically reflect the nutritional state of an individual [7,10,11,12]. For example, unchanged plasma/serum Zn concentrations were observed with intakes as low as 2.8 mg kg^−1^ to as high as 40 mg kg^−1^, showing the limitation of plasma Zn status to reliably present the dietary Zn intake [13,14]. Serum Zn levels tend to rise and then fall after a meal [15].

While urinary Zn decreases under severe Zn deficiency [16], the precise evaluation of Zn status in mild to moderate Zn deficiency is challenging, as studies with this biomarker give inconsistent and conflicting results [6]. Urinary Zn has been shown to be a poor indicator of early stage Zn deficiency [14,16].

Recently, a few studies provided some evidence to support the effectiveness of hair Zn concentrations in predicting the Zn status of individuals [17,18,19]. The hair Zn biomarker has some advantages, such as low cost and viability; however, hair zinc still lacks sufficient evidence towards validity as a method to assess Zn status [8,20]. Finally, the reliability of all currently used biomarkers under infection and inflammatory conditions is intricate [6,7]. Plasma Zn concentration can fall as a result of factors not related to Zn status or dietary Zn intake, i.e., infections, inflammation, trauma, and stress [7].

An indicator that truthfully represents Zn status under various physiological conditions in humans is still missing [7]. The role of Zn in various processes and pathways in the body is multifaceted, and this may indicate that one single biomarker may never be sufficiently sensitive and that we need to use a spectrum of Zn biomarkers to be able to precisely differentiate between various Zn deficiency states.

Emerging biomarkers are defined as ‘biomarkers for which there is some theoretical basis of a relationship to zinc intake or status, but the testing is insufficient to confirm the relation’ [8]. Currently, nail Zn concentration, Zn-dependent proteins, oxidative stress, DNA integrity, and taste acuity are placed in the group of emerging biomarkers [8]. However, for many of the newly identified biomarkers, insufficient evidence is available to demonstrate their true potential and further studies are needed to confirm which ones can be used as biomarkers of Zn status. In this review, we provide the existing evidence to show that the LA:DGLA ratio is likewise an emerging biomarker of Zn status that needs to be assessed further.

## 3. Delta 6 Desaturase: Structure, Regulation, and Function

Delta 6 desaturase (∆6 desaturase, D6D or Δ-6-desaturase) is a membrane-bound desaturase enzyme required for the synthesis of polyunsaturated fatty acids (PUFA). The enzyme is molecularly identical across all living organisms. ∆6 desaturase is widely expressed in human tissues, in the liver, the membrane of red blood cells, lung, and heart, with the highest levels being present in the brain [21,22].

**Figure 1:**
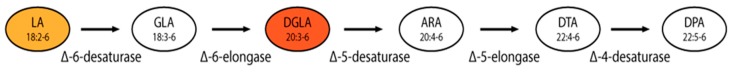
Desaturases involved in the biosynthesis of omega 6 polyunsaturated fatty acids. LA: linoleic acid; GLA: γ-linoleic acid; DGLA: dihomo-γ-linolenic acid; ARA: arachidonic acid; DTA: docosatetraenoic acid; DPA: docosapentaenoic acid. ∆6 desaturase is responsible for the formation of the carbon-carbon double bonds, and the function of an elongase is to lengthen fatty acid chains by the addition of two carbon units. LA (18:2-6) is desaturated by a ∆6 desaturase, introducing a D6 double bond into the substrate, giving γ-linolenic acid (GLA, 18:3-6). GLA is then elongated by a ∆6 elongase to dihomo-γ-linolenic acid (DGLA, 20:3-6). Modified from: Meesapyodsuk & Qiu, 2012 [25].

Linoleic acid (LA) is an essential omega 6 (*n* = 6) fatty acid that cannot be synthetized in the human body and must be obtained from the diet to ensure the appropriate development of various cells throughout the body [23]. It is the most abundant PUFA in human tissues [24]. LA is a metabolic precursor of dihomo-γ-linolenic acid (DGLA) (Figure 1). ∆6 desaturases are rate-limiting enzymes in the synthesis of PUFA, responsible for conversion of LA to DGLA, and the enzyme catalyzes the addition of a double bond at the sixth carbon-carbon bond position from the carboxylic acid end in fatty acids [22,25].

**Figure 2:**
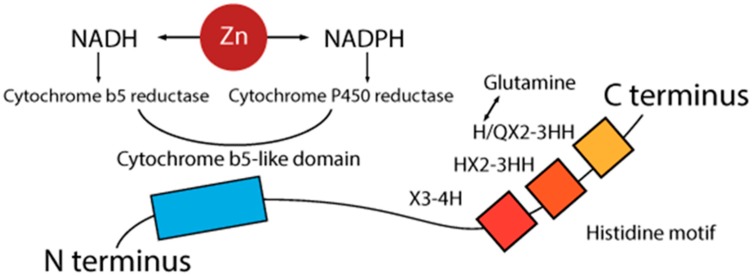
Schematic presentation of the structure of a ∆6 desaturase enzyme. A cytochrome b5-like domain is attached to the N-terminus and a histidine motif is located at the C-terminus. The histidine sequence is made of three histidine-rich motifs. The first histidine of the third motif is often replaced by glutamine. NADPH reductase has a Zn-dependent activity. NADH: nicotinamide adenine dinucleotide hydride; NADPH: nicotinamide adenine dinucleotide phosphate-oxidase.

The first ∆6 desaturase gene was cloned in 1993 from a cyanobacterium, *Synechocystis* [26]. Subsequently, desaturases have been identified and characterized from a wide range of species, and in 1997 the first eukaryotic ∆6 desaturase gene was cloned [27,28]. Mammalian ∆6 desaturase encoded gene is a protein made of a cytochrome b5-like domain, attached to the N-terminus of the main desaturation domain, and a histidine motif, located on a desaturation domain at the C-terminus [29]. The histidine sequence is made of three highly conserved histidine-rich motifs, i.e., HX3-4H, HX2-3HH, and H/QX2-3HH (Figure 2). The first histidine of the third motif is commonly substituted by glutamine [27]. The conversion of glutamine back to histidine results in the loss of activity, suggesting that the process might be very important not only for the structural configuration of desaturases, but also for their activity [30]. The gene coding for Δ6 production is located on human chromosome 11 (11q12.2-13.1), and is made of 12 exons and 11 introns [31].

To date, there has been limited information available on how the expression of the ∆6 desaturase is regulated. Some scientists believe that the regulation is achieved by the feedback control of the transcriptional regulation of fatty acid desaturase genes, mediated through signaling pathways activated by sensors embedded in cellular membranes, in response to environmental factors [32]. There is also some evidence showing that ∆6 desaturase enzymatic activity may be determined by tissue-specific mechanisms that involve both pre- and post-translational events [25].

NADPH reductase is important in the action of the ∆6 desaturase and is a Zn-dependent enzyme [33,34,35]. Typically, the Zn atom is bound to three or four ligands, which are composed of amino acids residues with histidine being the most frequent, followed by glutamic acid, aspartic acid, and cysteine [36]. Finally, the mutation of the cytochrome b5 domain is critical for the activity of ∆6 desaturases [37,38].

## 4. The Role of Zn in the Regulation of ∆6 Desaturase Activity and Fatty Acid Metabolism

Zn is an essential component of many enzymes and constitutes a part of their prostetic group [39]. It is present in DNA and RNA polymerases, dehydrogenases, and desaturases, regulating their functions via its catalytic, structural, or regulatory role. Over the years it has been noted that Zn is an important co-factor for the metabolism of fatty acids [40]. Zn is also necessary for at least two stages in essential fatty acid (EFA) metabolism; the conversion of linoleic acid to γ-linolenic acid, and the mobilization of dihomo-γ-linolenic acid (DGLA) to arachidonic acid [41]. Zinc has an effect on ∆6 desaturase itself [41], and affects linoleic acid absorption [42]. Once it was identified that Zn and essential fatty acid deficiencies give similar symptoms, a close association between fatty acid metabolism and Zn status was proposed [41,43,44].

Over the years, a number of studies have demonstrated an effect of Zn deficiency on the metabolism of essential fatty acids by impaired ∆6 desaturation activity [34,42,44,45,46]. On the other hand, there are a few studies completed in early 1990 that showed no role of Zn in fatty acid desaturation [47,48,49,50,51].

The effect of Zn deficiency on ∆5 and ∆6 activity was initially investigated by a number of groups in the early 1980s [42,45,52]. The findings were consistent, proving the reduced activity of ∆6 desaturase during Zn deficiency. Ayala and Branner [42] used male weaning Wistar rats and examined the influence of Zn on desaturating enzymes of liver and testes microsomes and their impact on fatty acid and lipid alterations of the tissues. The rats were fed Zn-adequate (55 ppm of Zn) or Zn-deficient diets (1.2 ppm of Zn) for 60 days. The progress of the effect of Zn deficiency was notable; Zn deficiency induced a decrease of essential fatty acids of the linoleic family in plasma after only 18 days, which indicates that Zn deficiency causes a rapid change in desaturase activity [42]. The activities of both desaturases were affected by Zn deficiency, but to a different degree. The same level of Zn deficiency caused a 45% reduction in ∆6 activity, while ∆5 was almost completely attenuated [42].

Similar findings were provided by Cunnane and Wahle [53] when it was shown that Zn modulates linoleic acid metabolism in rat mammary glands, modifying the ∆6 desaturation of microsomes. Specifically, 38 Sprague-Dawley rats were fed either a purified Zn-supplemented or a Zn-deficient diet for six weeks. The effects of Zn deficiency on the fatty acid composition of plasma lipids and microsomes of liver, intestine, and testes were studied. Among the polyunsaturated fatty acids, DGLA was significantly reduced by the Zn-deficient diet. Interestingly, the activity of ∆6 activity in liver microsomes was decreased by 25%, while the ∆5 desaturation was reduced by 53% in Zn-depleted rats. In addition, hypertriglyceridemia was observed in the serum of Zn-deficient rats. This study demonstrated that Zn supplementation returned serum triglycerides to normal levels, which shows a strong physiological interaction between Zn and EFAs (essential fatty acids) and confirms that Zn deficiency is responsible for the defects in desaturation [54].

Ten years later, studies completed by Eder and Kirchgessner [44,55,56] provided somewhat contradictory results, demonstrating that Zn deficiency does not affect ∆5 and ∆6 desaturation. The experiments were conducted using various types of dietary fats, including coconut, sunflower, or linseed oil. The proposition was that the activities of ∆5 and ∆6 desaturase depend on the type of dietary fat consumed. Diets rich in fats with high levels of polyunsaturated fatty acids suppress activities of desaturases, while fat free diets significantly raise the activities of these desaturases.

Later in 1995, the authors suggested that one reason for the contradiction between the findings might be the experimental design used in the studies, where the effects of Zn deficiency on desaturase activity was misperceived by a low food intake. After that, the role of Zn in desaturase activity was examined by a serious of experiments with Zn-deficient rats using a force-feeding technique that ensures identical food intake [44]. ∆5 and ∆6 desaturation was investigated in the presence of Zn deficiency in force-fed rats by previously raising the levels of enzymes by feeding a fat-free diet [44]. Zn-deficient rats fed a diet consisting of 5% safflower oil had lower levels of total PUFA than the corresponding rats fed a Zn-adequate diet. The authors clearly demonstrated the role of Zn in ∆5 and ∆6 desaturation in subjects with adequate food and energy intake. Similarly, in subjects with a low-fat intake (fat-free diets), the effect of Zn deficiency on ∆6 desaturation activity was even more pronounced, with a significantly lower activity of the enzyme being observed [44].

In 1999, Waldhauser and colleagues [57] looked at the ratio between *n*-3 PUFA and *n*-6 PUFA in zinc-deficient animals. Four groups of rats were fed zinc-deficient (0.5 mg Zn kg^−1^) or zinc-adequate (45 mg Zn kg^−1^) diets with either olive oil or linseed oil as the source of fat. To ensure an adequate food intake, the rats were force-fed by gastric tube over a period of 13 days. The study confirmed that Zn deficiency influences the metabolic balance between *n*-3 and *n*-6 PUFA, whereas saturated and MUFA (monounsaturated fatty acids) seem to remain unaffected by Zn deficiency. In the rats that were fed linseed oil, zinc deficiency caused a marked increase in the ratio between *n*-3 and *n*-6 polyunsaturated fatty acids in liver phospholipids, particularly in phosphatidylcholine. In contrast, in the rats that were fed olive oil, Zn deficiency had only slight effects on the fatty acid composition of the liver phospholipids. Therefore, this study confirms the previous results demonstrating that the effects of Zn deficiency on lipid metabolism may be influenced by the type of dietary fat. However, it must be noted that only hepatic ∆6 desaturase enzymatic activity may be reliant upon the composition of dietary fat [58]. This is not applicable to all other tissues. While, the consumption of an essential fatty acid-deficient diet is paralleled by a similar increase in the hepatic abundance of ∆6 desaturase mRNA and the increase in hepatic ∆6 desaturase activity [21], ∆6 desaturase activity was very low in non-hepatic tissues [58,59].

It seems that the potential role of dietary fat on desaturase activity, under relevant conditions, is only related to hepatic tissue. Similarly, in situations when EFA deficiency is of dietary origin, there is an increased attempt to synthetize more linoleic acid, so ∆6 desaturase activity is increased. However, when EFA deficiency is metabolic (as in Zn deficiency) ∆6 activity is inhibited [60]. Finally, increased ∆6 desaturase activity will not necessarily lead to the elevated metabolizing of linoleic acid and its conversion to DGLA. Below, we summarize the findings that confirm the interaction between Zn deficiency and the metabolism of linoleic acid via desaturase enzymes:

1. Zn may have a role in the absorption of linoleic acid. Lower levels of Zn produce lower levels of linoleic acid [60].
2. Zn has a role in relation to the NADH-NADPH cycle (Figure 2) [35].
3. Cytochrome P-450 activity is significantly reduced under Zn deficiency [34].
4. Zn deficiency reduces the availability of linoleic acid metabolites γ-linolenic and arachidonic acid [52,61].
5. Zn deficiency decreases the mobilization of DGLA from tissue stores [62].
6. Zn is needed in the formation of GLA and in the mobilization of DGLA [41].
7. EFA supplementation worsens the effect of Zn deficiency [43,52,63].
8. Zn deficiency decreases the esterification of essential fatty acids into phospholipids [46].
9. During Zn deficiency, linoleic acid accumulates in tissues when EFA supplements are administered [63].
10. Zn deficiency results in a higher concentration of linoleic and a lower concentration of arachidonic acid in tissue phospholipids [34].
11. Zn-deficient subjects have an increased β-oxidation of linoleic acid, resulting in decreased amounts of linoleic acid available to be metabolized into arachidonic acid [64].
12. The most important EFA functions are carried out by molecules downstream of GLA [41].
13. In animals exposed to diets deficient in essential fatty acids, the characteristic symptoms develop much more rapidly if the diets are also deficient in Zn [43,65].
14. The inhibition of the desaturases by Zn deficiency is so strong that it causes a more rapid decline in tissue arachidonic acid and docosahexaenoic acid than does the direct dietary deficiency of all the omega 6 or omega 3 polyunsaturated fatty acids [64].
15. Enzymes involved in prostaglandin synthesis are also Zn-dependent, and defects in prostaglandin synthesis are observed under Zn deficiency [52].

In summary, Zn has both a direct role in the modulation of desaturase activities involved in the fatty acid metabolism and an indirect effect on fatty acids by influencing their absorption, oxidation, and incorporation [34]. Zn deficiency causes inconsistencies in the ratio of desaturase substrates and products, and linoleic acid (LA) and dihomo-γ-linolenic acid (DGLA), respectively (Figure 1). The most important EFA functions are carried out by molecules downstream of GLA. The ∆6-catalyzed step required for the transformation of LA to DGLA is generally the highest flux pathway, so an elevation in the LA:DGLA ratio could be a sensitive marker for Zn deficiency.

## 5. The LA:DGLA Ratio as a Biomarker of Zn Status, Current Evidence

In 2014, the concept of the essential role of Zn for ∆6 desaturase activity was reinvented. For the first time, Reed et al. [66] tested and implemented a previously unexplored biomarker of zinc status related to erythrocyte Δ6 desaturation, the LA:DGLA ratio. By using the chicken (*Gallus gallus*) as a model, the authors evaluated the sensitivity of the erythrocyte LA:DGLA ratio to changes in supplemental Zn intake. A significant negative correlation was found between dietary Zn deficiency and the LA:DGLA ratio.

The *Gallus gallus* has a similar membrane fatty acid composition to mammals [67] and is highly sensitive to dietary Zn manipulations [66,68,69,70], which makes it a potentially ideal animal model for exploring changes in the production of essential fatty acids in relation to Zn nutrition. In the original study, birds were fed either a Zn-adequate control diet (42.3 μg Zn g^−1^) or a Zn-deficient diet (2.5 μg Zn g^−1^). Diets had identical FA (fatty acids) content/profile. The body weight, feed consumption, Zn intake, and serum Zn concentrations of the birds were measured weekly, showing higher values of all parameters in the Zn control versus the Zn-deficient diet group of birds (*p* < 0.05). There was a relative increase in gene expression of the cytokines: tumor necrosis factor alpha (TNF-α), interleukin 1 beta (IL-1β), and interleukin-6 (IL-6) in the control group. Other assessed parameters included metal transporters (i.e., ZnT1, ZnT5, ZnT7, Zip6, Zip9); transcription factor: nuclear factor kappa B (NF-κB); brush border enzymes: aminopeptidase, sucrose-isomaltase, Na+K+ATPase, sodium glucose transport protein 1 (SGLT-1), and binding metallothionein-4 protein (MT4). These parameters were found to not be significantly different between the groups. However, the expression of hepatic Δ6 desaturase was significantly higher in the control group (*p* < 0.001). Accordingly, the LA:DGLA ratio was noticeably elevated in the low Zn compared to the control Zn group (22.6 ± 0.5% and 18.5 ± 0.5% *w*/*w*, respectively, *p* < 0.001). This study demonstrated that the erythrocyte LA:DGLA is able to differentiate zinc status between zinc-adequate and zinc-deficient subjects. Furthermore, variations in the LA:DGLA ratio were noticeable within seven days, signifying that this biomarker can show changes in the dietary Zn status quickly and that it may be able to detect early stages of Zn deficiency that usually, due to the lack of obvious signs and symptoms, pass unrecognized.

This proposed biomarker has been evaluated further in humans [71]. A study was completed on healthy human volunteers, 25–55 years of age. The content of plasma phospholipid LA, DGLA, and changes in the LA:DGLA ratio were compared to the dietary Zn intake and plasma Zn status in human subjects. Participants were separated into two groups based on dietary Zn intakes, assessed using three 24-h recall questionnaires provided on three non-consecutive days. There were no statistically significant differences in the dietary intake of LA and PUFA among the groups of participants. Plasma phospholipid fatty acid analysis was conducted by gas chromatography, and plasma analysis of minerals was conducted by atomic absorption spectrometry.

In addition, the study assessed correlations of plasma Zn and the LA:DGLA ratio with various biochemical, anthropometrical, and hematological parameters. It was shown that while the plasma Zn concentrations of participants remained unchanged (most likely due to the good homeostatic regulation), there was a statistically significant difference in DGLA production and the LA:DGLA ratio between the groups (*p* < 0.05). The concentration of plasma DGLA was decreased and the LA:DGLA ratio was increased in people with lower dietary Zn intakes. Besides, docosatetraenoic acid (22:4n-6; D5D) was also lower in the group of people with lower dietary Zn intake.

Finally, the efficacy of the LA:DGLA ratio to predict the Zn status of subjects consuming a wheat-based diet, a diet more representative of a diet of the target Zn-deficient populations, was recently evaluated in vivo by using the *Gallus gallus* model [72]. Two groups of birds (*n* = 15) were fed two different diets, a “high-Zn” diet (46.5 ppm Zn) and a “low-Zn” diet (32.8 ppm Zn), for six weeks. Dietary zinc intake, body weight, serum zinc, and the erythrocyte fatty acid profile were assessed. Serum zinc concentrations were greater in the high-Zn group (*p* < 0.05). Similarly, the concentration of Zn in tissues (feather and nail) was higher in the high-Zn group of birds as opposed to the birds fed a low-Zn diet (*p* < 0.05). Duodenal mRNA expression of various Zn transporters (i.e., Zip4, Zip6, Zip9, ZnT1, Znt5 and Znt7) demonstrated a higher mean value in the tissues collected from the birds fed a low-Zn diet (*n* = 15, *p* < 0.05). The measurements of hepatic ∆6 desaturase expression showed significant differences between the groups, with a higher mean value in birds fed high-Zn diets. The LA:DGLA ratio was higher in the low-Zn group of birds at all time points measured (weeks 2, 4, and 6). Once more, the LA:DGLA ratio responded to changes in dietary Zn intake. Even though both groups of birds were fed Zn-deficient diets, with only 14 ppm differential in dietary Zn content, still the LA:DGLA ratio differentiated clearly between the groups, which demonstrates the sensitivity of the biomarker to change in accordance with dietary Zn intake.

## 6. Conclusions and Recommendations for Further Research

The evidence provided in this review demonstrate the potential of the LA:DGLA ratio to be used as an additional biomarker of Zn status in humans. To date, research shows that the LA:DGLA ratio corresponds to dietary Zn manipulations, both in animals and humans. This biomarker should be tested and evaluated further to illuminate its full potential. This review provides some evidence to justify the requirements for further research.

Well-controlled human dietary intervention trials are needed to examine the sensitivity of this biomarker in larger healthy cohorts, as well as in Zn-deficient populations. Hence, additional research is needed to elucidate any potential limitations of this biomarker, i.e., the effect of inflammatory conditions and infection states on this biomarker. Similarly, the enzymatic activity of hepatic desaturases should be compared to the expression and activity of ∆6 desaturases in non-hepatic tissues in order to determine the exact role of dietary fats in ∆6 desaturase activity. Additional studies are needed to clarify the potential impact of other nutrient deficiencies on the LA:DGLA ratio, in particular the effect of iron and copper deficiencies. The effectiveness of the LA:DGLA ratio, in relation to Zn status and Zn bioavailability over time, requires further investigation. The kinetics of other desaturase enzymes in relation to Zn intake should also be tested (i.e., Δ5 desaturase). Finally, the modifications of Zn-dependent proteins and genes at the main sites of Zn absorption, namely, in the small intestine, in relation to Zn intake and the LA:DGLA ratio need to be tested and evaluated further.
